# Supplementary material for: Climate warming has compounded plant responses to habitat conversion in northern Europe
Source: Nat Commun. 2022 Dec 19;13:7818. doi: 10.1038/s41467-022-35516-7 (PMC9763501; doi:10.1038/s41467-022-35516-7)
Supplement: Supplementary file 1 — Supplementary Information [file 41467_2022_35516_MOESM1_ESM.pdf]

SUPPLEMENTARY INFORMATION

Climate warming has compounded plant responses to habitat conversion in northern Europe

Alistair G. Auffret & Jens-Christian Svenning

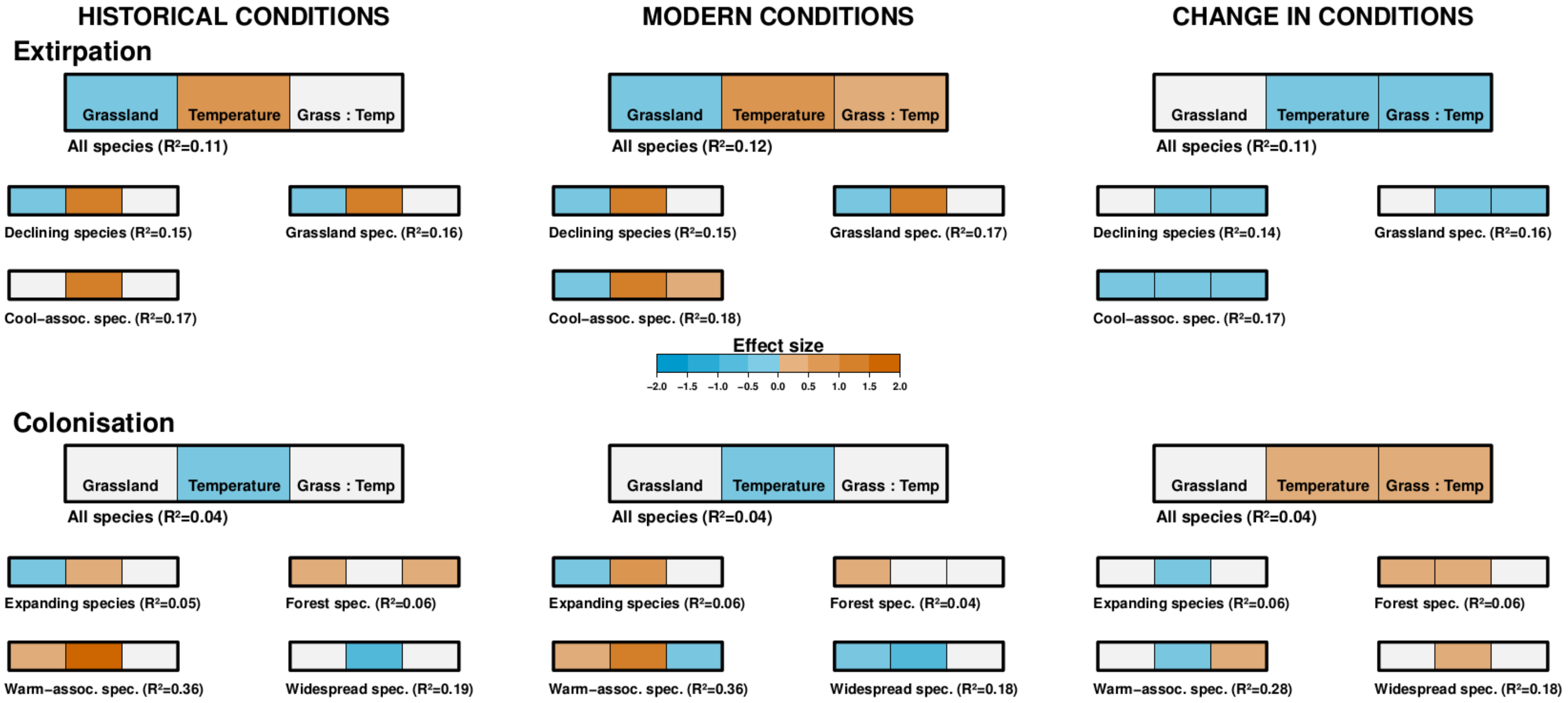

Supplementary Figure 1. The effects of land use and climate on plant community turnover. Blocks show the effect of grassland habitat, temperature and their interaction on the extirpation and colonisation of plant species in four Swedish provinces over a ~60 year time period. Model results are in batches according to whether the response variable was extirpation (fraction of species extirpated from a grid cell) or colonisation (fraction of the province's species pool that colonised a grid cell), and whether predictor variables were related to historical baseline conditions, modern conditions, or the change in conditions over time. Specifically, historical models considered annual mean temperature 1961-70 and the fraction of open habitat in grid cells according to maps from 1940s-50s, modern models considered annual mean temperature 2001-10 and the fraction of grid cells that were open both in the historical maps and the 2018 land cover map (grassland retention). The change models considered grassland abandonment (fraction of grid cells that were open in historical and forest in modern maps), the change in mean annual temperature between 1961-70 and 2001-10. Large blocks within each batch present results for all species, while smaller blocks show model outputs for ecologically-relevant subsets of species. For the colonisations in the forest specialist subgroup of species, grassland predictors were replaced by historical and modern forest cover (historical and modern conditions, respectively), while grassland abandonment (i.e. forest gain on former grassland) was retained for the change model. Colours indicating direction and strength of effect are only shown for predictors where $P<0.05$. R2 values refer to marginal R2, relating to the variance explained by fixed factors. Full outputs are available in Supplementary Data 6.

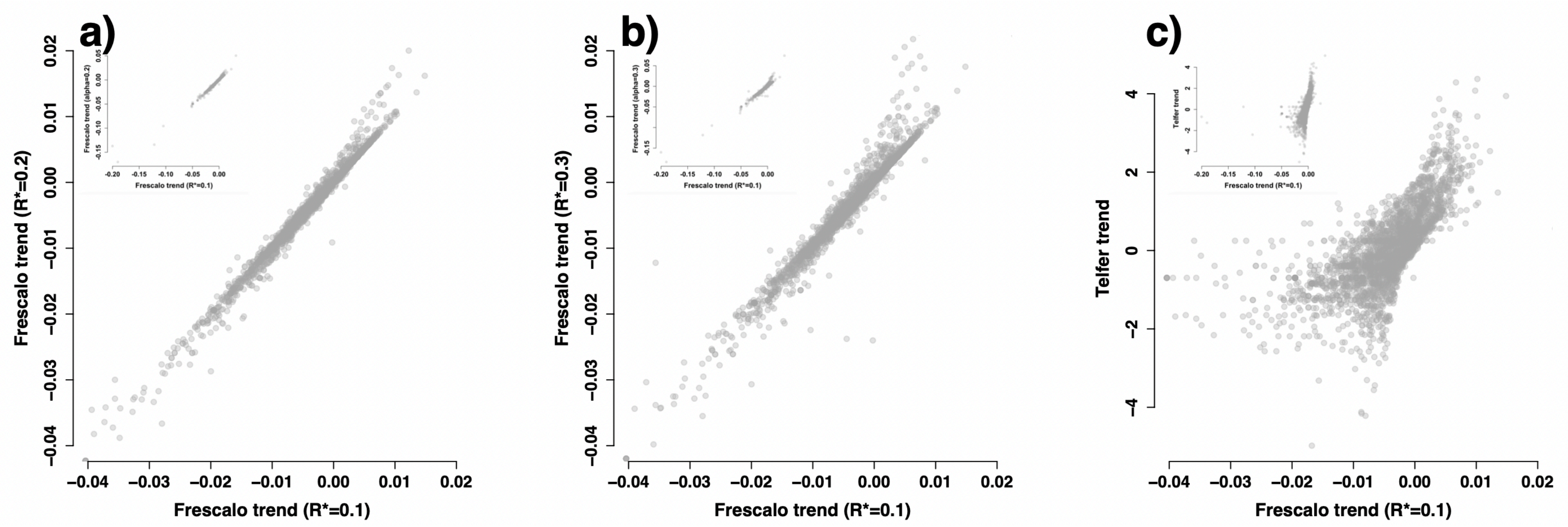

Supplementary Figure 2. Relationships between different estimations of species regional distribution change. Scatterplots of distribution change metrics for each species (grey points), comparing the main calculation (Frescalo with *R** value of 0.1) with metrics calculated using: a) Frescalo with *R** value of 0.2; b) Frescalo with *R** value of 0.3; and c) Telfer metric. Main plots have truncated axes to more clearly show the relationships, with inset figures displaying the full data range.

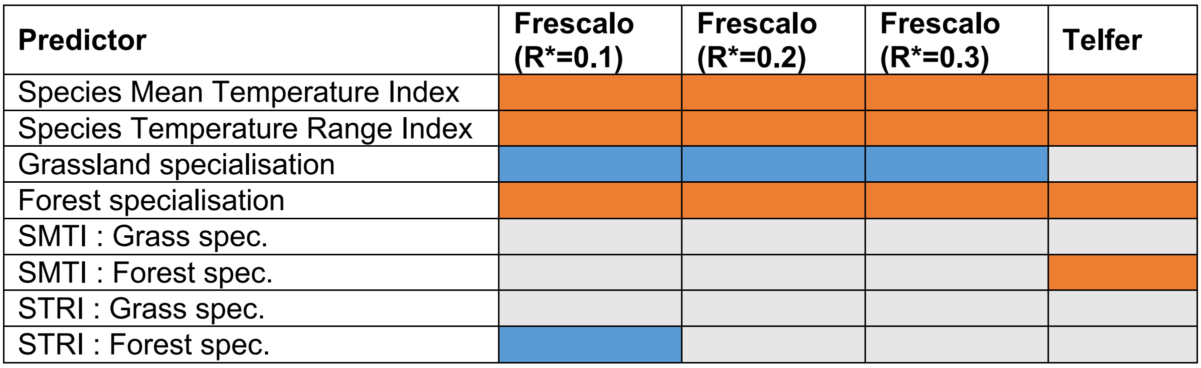

Supplementary Figure 3. Comparison of model outcomes relating correlates of different estimates of regional distribution change. Signals of the effects of predictor variables (orange = positive, blue = negative, grey = confidence intervals include zero) on alternative metrics of regional distribution change, showing overall consistent results. Full model outputs are found in Supplementary Data 8.

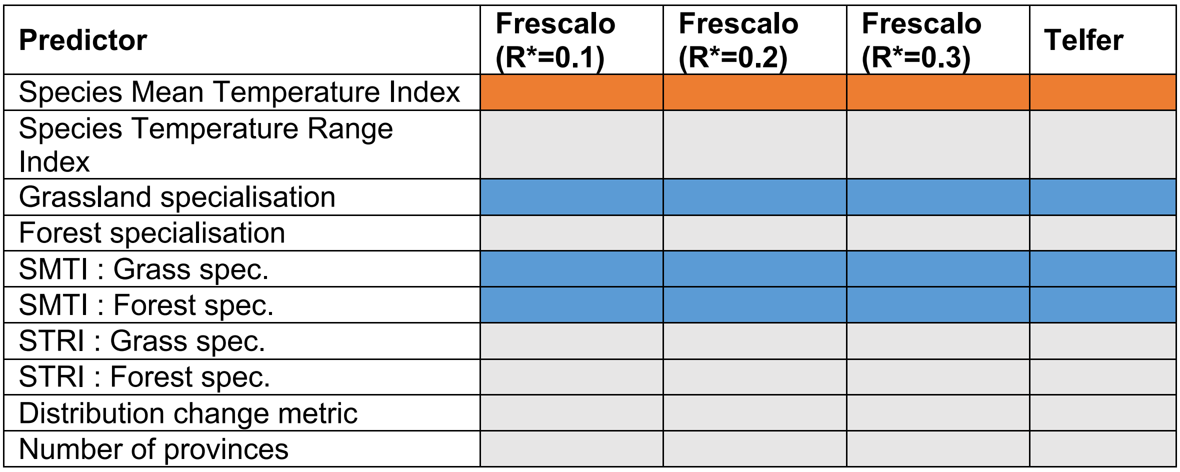

Supplementary Figure 4. Comparison of model outcomes relating correlates of species' shifts in climatic space. Signals of the effects of predictor variables (orange = positive, blue = negative, grey = confidence intervals include zero) on shifts in climatic space according to reference period temperatures (negative values of the response relate to shifts to on average relatively cooler, northerly distributions and *vice versa*), using alternative metrics of regional distribution change as a control predictor variable, showing overall identical results. Full model outputs are found in Supplementary Data 9.
