# Supplementary material for: Climate warming has compounded plant responses to habitat conversion in northern Europe
Source: Nat Commun. 2022 Dec 19;13:7818. doi: 10.1038/s41467-022-35516-7 (PMC9763501; doi:10.1038/s41467-022-35516-7)
Supplement: Supplementary file 3 — Description of Additional Supplementary Information [file 41467_2022_35516_MOESM3_ESM.pdf]

DESCRIPTION OF SUPPLEMENTARY FILES

File name: SupplementaryData1-9.xls

Description: Spreadsheet file containing 10 tabs. Readme: Information regarding the supplementary data;

Supplementary Data 1: Processed data containing species' distribution change estimates, and climate and habitat associations;

Supplementary Data 2: Model output from analysis of Supplementary Data 1;

Supplementary Data 3: Processed data containing species' climatic range information, and climate and habitat associations;

Supplementary Data 4: Model output from analysis of Supplementary Data 3;

Supplementary Data 5: Processed data containing grid square estimates of species turnover, and climate and land use variables;

Supplementary Data 6: Model outputs from analysis of Supplementary Data 5;

Supplementary Data 7: Fractions of species across gradient of habitat specialisation;

Supplementary Data 8: Model outputs from sensitivity analysis regarding the effects of species' climate and habitat associations on distribution change;

Supplementary Data 9: Model outputs from sensitivity analysis regarding the effects of species' climate and habitat associations on directional climatic range shifts.

File name: Supplementary Software.R

Description: R script used for analysing processed data in Supplementary Data 1, 3 and 5, to produce the model outputs in Supplementary Data 2, 4 and 6, respectively.
