# Supplementary material for: Climate warming has compounded plant responses to habitat conversion in northern Europe
Source: Nat Commun. 2022 Dec 19;13:7818. doi: 10.1038/s41467-022-35516-7 (PMC9763501; doi:10.1038/s41467-022-35516-7)
Supplement: Supplementary file 6 — Reporting Summary [file 41467_2022_35516_MOESM6_ESM.pdf]

nature portfolio

Corresponding author(s): Alistair Auffret

Last updated by author(s): Nov 30, 2022

Reporting Summary

Nature Portfolio wishes to improve the reproducibility of the work that we publish. This form provides structure for consistency and transparency in reporting. For further information on Nature Portfolio policies, see our Editorial Policies and the Editorial Policy Checklist.

Statistics

For all statistical analyses, confirm that the following items are present in the figure legend, table legend, main text, or Methods section.

n/a	Confirmed	
☐	☒	The exact sample size (*n*) for each experimental group/condition, given as a discrete number and unit of measurement
☒	☐	A statement on whether measurements were taken from distinct samples or whether the same sample was measured repeatedly
☐	☒	The statistical test(s) used AND whether they are one- or two-sided<br>*Only common tests should be described solely by name; describe more complex techniques in the Methods section.*
☐	☒	A description of all covariates tested
☐	☒	A description of any assumptions or corrections, such as tests of normality and adjustment for multiple comparisons
☐	☒	A full description of the statistical parameters including central tendency (e.g. means) or other basic estimates (e.g. regression coefficient) AND variation (e.g. standard deviation) or associated estimates of uncertainty (e.g. confidence intervals)
☐	☒	For null hypothesis testing, the test statistic (e.g. *F*, *t*, *r*) with confidence intervals, effect sizes, degrees of freedom and *P* value noted<br>*Give P values as exact values whenever suitable.*
☒	☐	For Bayesian analysis, information on the choice of priors and Markov chain Monte Carlo settings
☒	☐	For hierarchical and complex designs, identification of the appropriate level for tests and full reporting of outcomes
☐	☒	Estimates of effect sizes (e.g. Cohen's *d*, Pearson's *r*), indicating how they were calculated

Our web collection on statistics for biologists contains articles on many of the points above.

Software and code

Policy information about availability of computer code

Data collection	Raw data were processed using the open-source software R (versions 3.4 and 4.2).
Data analysis	We used the open-source software R (version 3.4 and 4.2), and the following libraries: sparta (0.2.19; which runs the Frescalo program, version March 2011) lme4 (version 1.1.29), performance (version 0.9), visreg (version 2.7), and raster (version 3.6.2). All software is fully cited in the reference list.

For manuscripts utilizing custom algorithms or software that are central to the research but not yet described in published literature, software must be made available to editors and reviewers. We strongly encourage code deposition in a community repository (e.g. GitHub). See the Nature Portfolio guidelines for submitting code & software for further information.

Data

Policy information about availability of data

All manuscripts must include a data availability statement. This statement should provide the following information, where applicable:

- Accession codes, unique identifiers, or web links for publicly available datasets
- A description of any restrictions on data availability
- For clinical datasets or third party data, please ensure that the statement adheres to our policy

Processed datasets used for analysis are available in the Supplementary files (Supplementary Data 1, 3 and 5). For raw data, historical and modern species observations are available from the Swedish species gateway ArtPortalen (https://artportalen.se; historical observations from Öland and Bohuslän scheduled for upload December 2022). Species temperature indices were taken from https://doi.org/10.6084/m9.figshare.8845832.v166, while habitat specialisation information

was extracted from the supplementary information of Tyler et al.13. Climate data are free to access from ftp://ftp.smhi.se/; contact kundtjanst@smhi.se for log-in details. Historical land-cover data are published at https://doi.org/10.17045/sthlmuni.4649854.v266, while modern land cover is published by the Swedish Environmental Protection Agency at https://metadatakatalogen.naturvardsverket.se/metadatakatalogen/GetMetaDataById?id=8853721d-a466-4c01-afcc-9eae57b17b39. The Swedish taxonomic database used for species name harmonisation is found at https://www.dyntaxa.se.

Human research participants

Policy information about studies involving human research participants and Sex and Gender in Research.

Reporting on sex and gender	N/A
Population characteristics	N/A
Recruitment	N/A
Ethics oversight	N/A

Note that full information on the approval of the study protocol must also be provided in the manuscript.

Field-specific reporting

Please select the one below that is the best fit for your research. If you are not sure, read the appropriate sections before making your selection.

☐ Life sciences ☐ Behavioural & social sciences ☒ Ecological, evolutionary & environmental sciences

For a reference copy of the document with all sections, see nature.com/documents/nr-reporting-summary-flat.pdf

Ecological, evolutionary & environmental sciences study design

All studies must disclose on these points even when the disclosure is negative.

Study description	We used historical and modern observations of plant species from plant atlases from four provinces in Sweden, covering 1232 5x5 km grid squares, to study how changes in species distributions over time are related to their climate and habitat associations, and how the climate and habitat changes that have occurred in each grid square affected the turnover that occurred over time. Our analysis was split into three stages: [1] Correlates of distribution changes of 1431 species for which distribution change could be calculated and for which climate and habitat associations were available; [2] Shifts in climatic space for 1680 species in 813 grid squares, and correlates of shifts for 1391 species for which climate and habitat associations were available; [3] Turnover (extirpations and presences) in all 1232 grid squares and for all 1701 species, related to the environmental changes that had taken place in that grid square over time.
Research sample	We aimed to analyse the effects of climate and land use change on Swedish plant species over 60 years, with a view to represent patterns of all species save those restricted to alpine and subalpine regions. For this reason we used data from four Swedish provinces (Bohuslän, Öland, Uppland and Medelpad). These are the only provinces in Sweden for which a historical (early 1900s) and a modern (early 2000s) plant atlas was published (for Öland publishing of the actual book is in process, but the data are complete), and for which historical (1940s-1960s) maps are available. All atlases contain observation records derived from inventories carried out with the objective of documenting the distributions of plant species within the province. Observations are assigned to the 5x5 Swedish national grid, and within these grids, we also calculated mean annual temperature for 1961-1970, 1961-1990, 2001-2010, using the Swedish meteorological and hydrological institute's database of modelled daily values (resampled from the database's 4x4 km grid). Historical land cover was based on published digitalisations of the 1940s-1960s Economic map of Sweden, that uses the same 5x5 km grid. Modern land cover was taken from the 2018 land cover map published by the Swedish Environmental Protection Agency, with categories harmonised to match the historical maps. Species' habitat and climate associations were taken from published studies. Full references and/or URLs are available in the main text.
Sampling strategy	We used observations for all species and all grid squares for which data were available, with the exception of analysis stage [2] see 'Data exclusions' below. With hundreds of 'sites' (grid squares), more than one thousand species, and four provinces, we consider that our data are sufficient to study the effects of climate and land-use change on species distributions.
Data collection	Observation data were largely collected by the authors of each plant atlas.
Timing and spatial scale	Historical observations were from the first half of the 20th century, with exact years of main inventories varying according to province (1901-1920 in Medelpad, 1910-1940 in Uppland, 1920-1945 in Bohuslän, 1910-1940 on Öland). Modern observations were based on inventories from 1975-2010, 1990-2010, 1990-2011, 2000-2020 in the four provinces, respectively. Together, the 1232 5x5 km grid squares cover 30800 square kilometres.
Data exclusions	Approximately one-third of grid squares were excluded from analysis stage [2]. This is because it is important when quantifying range shifts that the grid squares are all well-recorded (so that one can be more confident that a colonisation is a true colonisation). Therefore, grid squares were included that contained observations of at least 10% of the province’s observed species and at least 25% of the species found in the eight adjacent grid cells in each time period. Similar cutoffs are used for existing studies, cited in the main text.

Reproducibility	This was not an experimental study.
Randomization	We did not employ randomisation in this observational study. However, we did control for a number of factors in our models. In all analyses stages we included a measure of observer effort, either in the calculation of distribution change [analysis 1 and 2] or as an estimate of effort [analysis 3]. In analysis [3] we also controlled for spatial autocorrelation, latitude and microclimatic variation, which might all affect how land use and climate can affect species turnover.
Blinding	Blinding was not relevant for our study. We had no preconceptions about which species or grid squares might respond to environmental change and how.

Did the study involve field work? ☐ Yes ☒ No

Reporting for specific materials, systems and methods

We require information from authors about some types of materials, experimental systems and methods used in many studies. Here, indicate whether each material, system or method listed is relevant to your study. If you are not sure if a list item applies to your research, read the appropriate section before selecting a response.

Materials & experimental systems

n/a	Involved in the study
☒	☐ Antibodies
☒	☐ Eukaryotic cell lines
☒	☐ Palaeontology and archaeology
☒	☐ Animals and other organisms
☒	☐ Clinical data
☒	☐ Dual use research of concern

Methods

n/a	Involved in the study
☒	☐ ChIP-seq
☒	☐ Flow cytometry
☒	☐ MRI-based neuroimaging
